# Voyages in Vacation.—XIII

**Published:** 1888-12-22

**Authors:** 


### VOYAGES IN VACATION.—XIII.

BY ONE IN SEARCH OF RESTFUL RESTORATIVES.

*(Continued from p. 172.)*

### ROYAL CARRIAGES—RAILWAYS—MOSCOW.

ONE very interesting exhibition of carriages is to be found in the Imperial Coach-houses, the walls of which are hung with tapestry of no small value. This tapestry, most of which came from the *Gobelins*' manufactory, consists of many fine pieces, the subjects being many of them sacred, is alone worth a visit. The collection of carriages is remarkable and interesting. Here will be found the sledge of Peter the Great, built with his own hands, and so regarded as the gem of the collection, it is placed in a glass case to preserve it as much and as long as possible. The windows are of horn (mica), and a large trunk is fastened behind, in which the Royal impedimenta was safely stowed away. One or two masquerade sledges, in various grotesque designs, by Italian builders, and used for Carnival purposes, cannot fail to amuse and interest. The state or gala carriages of Russian Czars and Empresses are truly magnificent. Some of the panels bear paintings by Watteau, Boucher, and Gravelot, nine of them are of gilt metal, several are made gorgeous with jewels, incrustations of mother-of-pearl, Spanish point, or jewelled and Imperial crowns, and the richest upholstery. The travelling and town equipages of the Court are also worthy of inspection, whilst a gruesome horror is given to the whole collection by the presence of the brougham in which Alexander II. met his death, the dynamite which exploded beneath it having shattered the seat, sides, and other portions in a terrible manner. The Imperial harness is also magnificent, though in the best of taste, and the stables, containing nearly 500 horses, are truly suggestive of kingly pomp and abundance.

The journey from Petersburg to Moscow is made by railway, the evening mail being the best train, and doing the distance in 14 hours. The fare is about £3 each way first-class, with an extra 5s. for a sleeping berth. As to the last, if travelling alone it is desirable to take two sections or berths, paying 10s., or if with companion £1, which payments secure a separate section in the first case and a whole compartment in the second. There is not much to be seen en route, the country being flat and uninteresting except for the occasional glances obtained of the villages, which consist of wretched hovels, or rather wooden sheds, with little or no comfort, space, or air, where cleanliness is difficult, and healthy living impossible. The stations are well built, the line is also fairly laid, and the roadway sound, whilst the refreshments at the stations are certainly the best we have ever had the good fortune to encounter on any railway in Europe or America. Our refreshment contractors might well take a lesson from the Russians who supply the stations on this railway. Some of the towns passed through on the journey are of fair size, but none have more than 30,000 inhabitants we understand. The impression made upon a thoughtful traveller by an inspection of a Russian village is appalling. He cannot credit how the poor, *i.e.*, the serfs, live. Indeed, they do not live at all, for to barely exist under such conditions must be difficult indeed. We are familiar with Scotch bothies, Irish cabins, and the worst of English tenement houses and rural cottages, but a Russian peasant's so-called house beats the record. Anything more repelling, miserable, or unhealthy, anything more condemnatory of the system of government which permits and tolerates it, or better calculated to debase and lower the population, cannot be conceived. Nihilism indeed; better go to Siberia or be shot from a gun than be condemned to the enforced miseries of the Russian poor.

As the train neared Moscow, the guards and attendants went out on the platform, keeping a sharp look-out in the direction where the Holy City was known to be. They evinced the greatest anxiety to see it the moment it came within range, for the reason, as we have since discovered, that every true Russian regards Holy Mother Moscow with religious fervour, and immediately it is seen, off go hats, heads are bowed low, prayers are said, and many crosses made with great reverence. It is the practice of the lower class of Russians to make this obeisance on first seeing the gold-covered domes of Moscow, which is said to contain nearly 2,000 churches and shrines. Arrived at the station, a perfect babel of chatter arose from the *Ischvostchiks*, or drivers, who literally swarmed, entering the train and attacking the passengers by gestures and signs whilst striving to forcibly possess themselves of their luggage, all being done in a most bewildering, not to say alarming fashion. Knutzé, our courier, was, however, more than a match for these noisy intruders, and we were soon safely seated in a comfortable carriage en route for our hotel. The pace was tremendous, and as the stones on the roadways were more numerous and more irregularly and roughly laid than in Petersburg, bad as that city is in these respects, the jolting and noise became almost maddening.

The distance from station to hotel proved considerable, and we thus gained a fair idea of the streets and houses. The majority of the former are narrow, ill kept, and uneven, whilst the houses appeared poor and unattractive when seen in the full blaze of a mid-day sun. The first distinct impression we were conscious of was the emphatically Eastern character of Moscow, as brought home to us by its quaint and many-coloured buildings, the varied and cosmopolitan character of the dresses worn by the people, the innumerable and glittering domes of the churches, the richly-illuminated shrines at every corner, each with a group of worshippers before it, the variety and oddness of the vehicles and harness, the display of Eastern wares of all kinds in the shops, and the evidence of great age with many varied architectural features which the buildings often presented. We were not so much disappointed as surprised by the poorness of much we thus witnessed—an impression, as we afterwards realised, which was greatly due to the searching character of the strong sunlight which penetrated every nook and corner of the city, and so brought the many imperfections and evidences of neglect and decay forcibly under the eye of the beholder. Later in the day, when we drove back from Sparrow Hill, as the sun was setting, we were all greatly impressed, even to silence, as the subdued light gave a peaceful and fascinating appearance to the whole city; the variety of colours and irregularity of design in streets and buildings pleasing the eye, and the general air of repose over Moscow at this hour making us realise how truly remarkable, and in many respects beautiful, the Holy City undoubtedly is. Altogether it was a novel experience, and the impressions we thus gained were not only pleasing, but so entirely new as to last one's lifetime. When viewed from Sparrow Hill, the spot where Napoleon first obtained a sight of Moscow, the city is very grand in many respects, but no true idea of the extent and variety of colour and design which it presents as a whole can be gathered by a stranger until he has ascended Ivan's Tower, which is situated within the Kremlin. Viewed from this vantage ground, all must acknowledge that Moscow leaves little else to be desired in the way of picturesqueness and effect. Indeed, many people hold to the belief that no other city in the world excels "Holy Mother Moscow," in novelty and beauty of appearance.

*(To be continued.)*

